# Quality of Evidence Supporting the Role of Supplement Curcumin for the Treatment of Ulcerative Colitis: An Overview of Systematic Reviews

**DOI:** 10.1155/2022/3967935

**Published:** 2022-02-25

**Authors:** Hongshuo Shi, Dan Wang, Wenqiang Chen, Yinghao Li, Guomin Si, Tiantian Yang

**Affiliations:** ^1^College of Traditional Chinese Medicine, Shandong University of Traditional Chinese Medicine, Jinan, China; ^2^Department of Traditional Chinese Medicine, Provincial Hospital Affiliated to Shandong First Medical University, Jinan, China

## Abstract

**Objectives:**

Curcumin is a potential complementary treatment for ulcerative colitis (UC). This overview systematically summarizes and evaluates the existing evidence of curcumin in the treatment of UC.

**Methods:**

Two researchers searched seven databases for systematic reviews (SRs)/meta-analyses (MAs) which are about randomized controlled trials (RCTs) on curcumin for UC. Two researchers use the Assessment of Multiple Systematic Reviews 2 (AMSTAR-2), the Risk of Bias in Systematic Reviews (ROBIS) scale, the list of Preferred Reporting Items for Systematic Reviews and Meta-Analyses (PRISMA), and the Grading of Recommendations Assessment, Development, and Evaluation (GRADE) system to assess the included SRs/MAs.

**Results:**

Seven published SRs/MAs were included in our study. According to the results of the AMSTAR-2 assessment, all SRs/MAs are considered to be of very low quality. According to the ROBIS evaluation results, no SR/MA has been assessed as a low risk of bias. According to the results of the PRISMA checklist assessment, no SR/MA has been fully reported on the PRISMA checklist. According to GRADE, a total of 19 outcome indicators extracted from the included SRs/MAs were evaluated. The quality of evidence was 10 moderate, 6 low, and 3 very low.

**Conclusions:**

Curcumin may be an effective and safe complementary treatment for UC. However, further standard and comprehensive SRs/MAs and RCTs are needed to provide an evidence-based medical rationale for this.

## 1. Introduction

Ulcerative colitis (UC) is a type of inflammatory bowel disease characterized by recurrent abdominal pain, diarrhea, and bloody pus. The lesions involve the colonic mucosa and submucosa [1]. UC can cause significant disturbance of colon inflammation homeostasis and severe damage to the intestinal barrier function, affecting millions of people worldwide. It cannot be cured completely; it must be managed for life [2]. Aminosalicylic acid, hormones, and immunosuppressive agents are mainly used in the clinical treatment of UC, but there are obvious adverse reactions. For example, mesalazine is a first-line drug for the treatment of UC, but long-term use can damage the liver and kidney function of patients [3]. In addition, drug resistance, dependence, and adverse reactions further limit the clinical efficacy of UC [4]. Due to ineffective long-term treatment, UC is likely to develop into colon cancer, and as many as 15% of patients may require the surgical removal of the colon in the late stage of the disease [5]. Therefore, many UC patients as well as clinicians and researchers are increasingly considering complementary and alternative medicine options [6].

Curcumin is a yellow bioactive polyphenol compound extracted from the root of the turmeric plant (*Curcuma longa*). It has a wide range of physiological and pharmacological activities, including anti-inflammatory, antioxidant, anticancer, neuroprotective, and antidiabetic [7]. The drug is generally considered by the FDA to be safe and inexpensive [8], and it has been extensively studied in various diseases [9]. Curcumin has become quite common as a complementary therapy for UC [10]. Its anti-inflammatory effect is considered the most relevant mechanism by blocking I*κ*B kinase to inhibit NF-*κ*B, thereby inhibiting proinflammatory cytokines (IL-1, IL-6, and TNF-*α*) expression [11].

In the past few years, many systematic reviews/meta-analyses (SRs/MAs) have been conducted to evaluate the potential therapeutic benefits of curcumin for patients with UC. SRs/MAs are considered the gold standard for assessing the effects of clinical interventions; however, high-quality SRs/MAs can provide reliable evidence, whereas low-quality SRs/MAs may instead mislead clinical decisions [12]. Therefore, there may be a gap between the evidence-based clinical implementation of curcumin and its actual implementation in real-world dynamics. Clinical decision-making requires a comprehensive overview of the available evidence to identify potential benefits and harms of interventions [12]. Therefore, the aim of our study was to critically assess the scientific quality of relevant SRs/MAs regarding curcumin for the treatment of UC through a systematic overview.

## 2. Materials and Methods

### 2.1. Research Methods

The SR/MA overview is based on the guidelines specified in the Cochrane Handbook [13], the Preferred Reporting Items for Systematic Reviews and Meta-Analyses (PRISMA) statement [14], and some high-quality methodological overviews [15, 16]. Literature search, literature screening, data extraction, and quality evaluation of related evaluation tools are done independently by two researchers. If there is any inconsistency, disagreements are resolved through consensus or discussion with an experienced third researcher.

### 2.2. Development of Inclusion and Exclusion Criteria

#### 2.2.1. Literature Inclusion Criteria

- Study Design: This overview only includes SRs/MAs from randomized controlled trials (RCTs) of curcumin in the treatment of UC
- Study Participants: This study includes subjects who have been clinically or radiologically diagnosed with UC according to national or international standards, regardless of age, race, or gender
- Study Intervention: The experimental group was supplemented with curcumin on the basis of conventional treatment (CT), and the control group was treated with CT or CT supplemented with a placebo
- Study Outcome: (1) The clinical remission is defined as follows: Clinical Activity Index (CAI) score ≤4; Ulcerative Colitis Disease Activity Index (UCDAI) ≤2 or<3; and Simple Clinical Colitis Activity Index (SCCAI) ≤2; (2) The clinical improvement, defined as a decrease in UCDAI by ≥3, decrease in partial Mayo score by ≥3, and SCCAI score by ≥3 points; (3) The endoscopic response, defined as Mayo score drop ≥1 point and mucosal appearance score drop ≥1 point; (4) The endoscopic remission, defined as baron endoscopic score 0/1, Mayo endoscopic score 0 or 1, and partial Mayo score ≤1; (5) Related inflammatory factors, including C-reactive protein (CRP) and Erythrocyte sedimentation rate (ESR); and (6) Safety profile, including adverse events

#### 2.2.2. Exclusion Criteria

Repeated publications, other overviews, network meta-analysis, narrative reviews, and conference abstracts were excluded.

### 2.3. Search Strategy

We searched 7 databases, including PubMed, Embase, Cochrane Library, CNKI, Wanfang Database, Chongqing VIP, and China Biomedical Literature Database from its establishment until December 15, 2021. The search strategy adopts a combination of MeSH terms and free words. We searched the above databases through the following key terms: curcumin, ulcerative colitis, systematic reviews, and meta-analysis. We also manually searched the references for related articles. The specific search strategy is modified according to different databases.  Table 1 provides the search strategy for the PubMed database.

### 2.4. Eligibility Assessment and Data Extraction

Document management software (Endnote X9, Clavirate Analytics, USA) is used to manage the retrieved articles. After deleting duplicates, researchers read the title and abstract to find potential SRs/MAs based on the inclusion and exclusion criteria then obtained full-text articles for further screening to determine eligibility. They then used the standardized data extraction form to extract the data independently. The following specific characteristics are extracted from each SR/MA: first author, year of publication, country, number of included studies, sample size, treatment intervention, control intervention, mode of administration, quality assessment methods, results, and main conclusions.

### 2.5. Quality Assessment

#### 2.5.1. Assessment of Methodological Quality

The Assessment System for Evaluating Methodological Quality 2 (AMSTAR-2) [17] scale was used to assess the methodological quality of the included SRs/MAs. It consists of 16 items, 7 of which are critical areas (2, 4, 7, 9, 11, 13, and 15). Each item is assessed using three assessment options: yes, partial yes, or no.

#### 2.5.2. Assessment of Risk of Bias

The risk of bias of the included SRs/MAs is assessed by the Risk of Bias in Systematic Reviews (ROBIS) [18]. The scale is completed in 3 stages to assess the overall risk of bias. The results are judged as “low,” “unclear,” or “high.”

#### 2.5.3. Assessment of Reporting Quality

The list of PRISMA [14] is used to assess the quality of each SR/MA report based on the following areas: (a) title, (b) summary, (c) introduction, (d) method, (e) result, (f) discussion, and (g) funding. It consists of 27 projects, with a focus on the reporting methods and results in a meta-analysis. Based on the completeness of the project information report, each project is considered “yes” (full report), “partial yes” (partial report), or “no” (no report).

#### 2.5.4. Assessment of Quality of Evidence

The Grading of Recommendations Assessment, Development, and Evaluation (GRADE) [19] system is used to assess the quality of the evidence of the included SRs/MAs, downgrading from five aspects: research limitations, inconsistencies, indirectness, imprecision, and publication bias.

### 2.6. Data Synthesis and Presentation

In this overview, an objective description is used. The characteristics and results of each SR/MA and the evaluation results of AMSTAR 2, ROBIS, PRISMA, and GRADE are reported in the form of a list.

## 3. Results

### 3.1. Results on Literature Search and Screening

**Figure 1:**
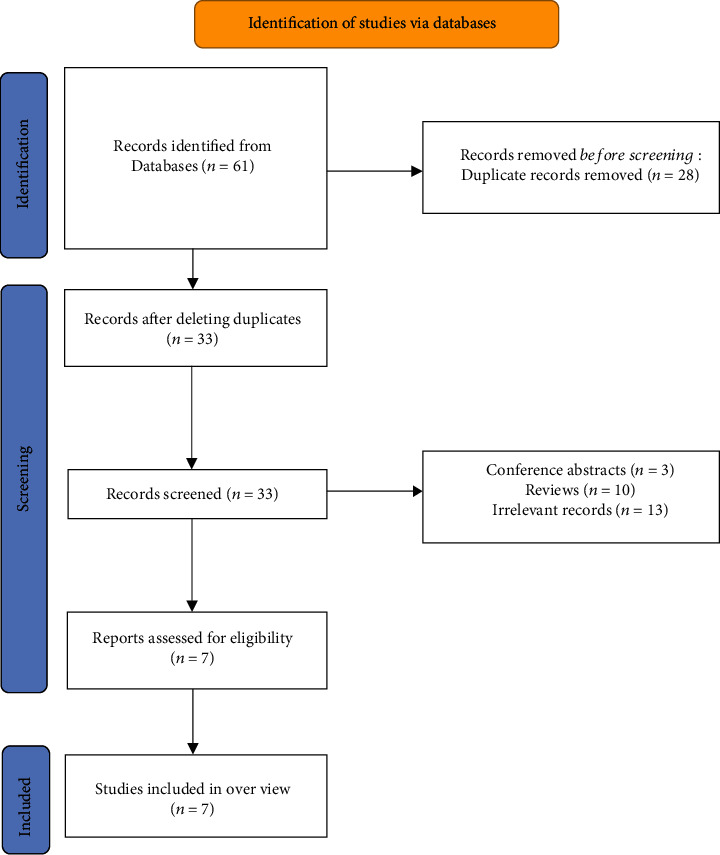
The flowchart of the screening process.

A total of 61 articles were retrieved through these seven literature databases, and 28 duplicate articles were deleted. We filter by the title and abstract of the literature and finally obtained 7 studies for full-text screening. After evaluation according to the inclusion and exclusion criteria, we finally included these 7 studies from the literature (Figure 1).

### 3.2. Description of Included SRs/MAs

Seven SRs/MAs [20–26] published from 2018 to 2021 were included. Of these published SRs/MAs, two are from China [25, 26], two from the United States [20, 23], and the remaining three from Brazil [22], Iran [21], and Greece [24]. Among them, 6 SRs/MAs were published in English [20–25], and one was published in Chinese [26]. The number of RCTs included in each SR/MA ranges from 2 to 7, and the sample size of a single study ranges from 104 to 380. The intervention of the treatment group was CT supplement curcumin, and the control group was treated with CT and a placebo. CT includes sulfasalazine and 5-aminosalicylic acid. The details of the SRs/MAs included are shown in Table 2.

### 3.3. Results on SR/MA Quality Assessment

#### 3.3.1. Methodological Quality Assessment

The AMSTAR-2 assessment breakdown for each review is shown in Table 3. Since more than one critical area is missing in the remaining SRs/MAs, the methodological quality of all SRs/MAs was assessed as very low. The method restriction comes from the following item: item 2 (only 1 SR/MA [24] has registered the protocol), item 7 (none of the SRs/MAs provides a research exclusion list), and item 15 (only 2 SRs/MAs [23, 25] conducted publication bias studies or discuss their impact on SR/MA).

#### 3.3.2. Risk of Bias of the Included SRs/MAs

The risk of bias for all SRs/MAs in the first stage (assessment of relevance) and Domain 1 (research eligibility criteria) of the ROBIS scale evaluation was assessed as low risk. Domain 2 evaluated the identification and selection of research, and 4 of the SRs/MAs [20, 21, 23, 24] were assessed as low risk. In Domain 3 (data collection and research evaluation), 3 SRs/MAs [20, 21, 23] were assessed as low risk of bias and none of the SRs/MAs is assessed as low risk in Domain 4 (synthesis and discovery). Phase 3 assessed the risk of bias in the review, and the 7 SRs/MAs had a low risk of bias. The evaluation details of the included SRs/MAs on the ROBIS scale are shown in Table 4.

#### 3.3.3. Report Quality

The results of the PRISMA inventory evaluation are shown in Table 5. 20 out of 27 items have a “yes” answer rate of more than 70%, and this shows that the report is relatively complete. However, there are some reporting deficiencies in other projects. Items 5 (protocol and registration), Items 15 (methods: risk of bias across studies), Items 16 (methods: additional analyses), and Items 22 (results risk of bias across studies) are inadequately reported (“yes” response rate is less than 50%).

#### 3.3.4. Evidence Quality

Table 6 shows the results of GRADE evaluation including SR/MA-related outcome indicators. The 7 SRs/MAs included 19 outcome indicators related to the efficacy and safety of curcumin on UC. In the evaluation results based on the outcome indicators, the quality of evidence was 10 moderate, 6 low, and 3 very low. Imprecision (*n* = 15) was the most common downgrade factor, followed by publication bias (*n* = 12), inconsistency (*n* = 5), risk of bias (*n* = 0), and imprecision (*n* = 0).

### 3.4. Summary of Results of the Included Studies

The result indicators extracted from the included studies are listed in Table 6.

#### 3.4.1. Clinical Remission and Improvement Rate

6 SRs/MAs [20, 22–26] reported the clinical remission rate, of which 5 SRs/MAs [20, 22, 23, 25, 26] showed that curcumin can significantly increase the clinical remission rate of UC patients. 5 SRs/MAs [20, 22, 23, 25, 26] reported the clinical improvement rate, of which 4 SRs/MAs [20, 23, 25, 26] showed that curcumin can significantly improve the clinical improvement rate of UC patients.

#### 3.4.2. Endoscopic Remission and Improvement Rate

One SR/MA [20] showed that curcumin can significantly increase the summary rate of endoscopic improvement and remission, and 2 SRs/MAs [23, 25] reported that curcumin can significantly increase the endoscopic improvement rate; in addition, 3 SRs/MAs [23, 25, 26] reported curcumin can significantly improve the endoscopic remission rate.

#### 3.4.3. Inflammatory Factors

One SR/MA [21] showed that curcumin can significantly reduce CRP and ESR levels.

#### 3.4.4. Adverse Events

Although none of the SR/MA provides a quantitative comparison of adverse events between curcumin and the control group, there are 3 SRs/MAs [20, 22, 25] that narratively report that there is no significant difference in the occurrence of adverse events between the curcumin group and the control group, and no serious adverse events occurred.

## 4. Discussion

UC is a chronic disease characterized by local tissue damage, intestinal flora imbalance, and colon inflammation. With the exception of corticosteroids, conventional treatments for UC are very expensive. Therefore, supplementary treatments are needed [27]. At the same time, more and more related SRs/MAs have been carried out. However, the quality of these publications has not been evaluated so far. In this case, we integrated the published results of SRs/MAs to provide a comprehensive evidence-based summary of the results of the clinical application of curcumin to UC.

### 4.1. Summary of the Main Findings

This is the first overview of SR/MA to study the effectiveness and safety of curcumin in the therapy of UC, including 7 SRs/MAs published between 2018 and 2021. This indicates more and more attention is paid to the effectiveness and safety of curcumin treatment of UC.

According to the results of the AMSTAR-2 evaluation, the methodological quality of all SRs/MAs was assessed as very low, especially in projects 2 (protocol registration), 7 (exclusion list), and 15 (publication bias). Only 1 SR/MA [24] was registered for the meta-analysis protocol. When conducting SR/MA, protocol registration is very important, and protocol registration should be done when determining the topic selection, which helps reduce the possibility of selective reporting bias and ensures SR/MA is carried out in an orderly manner [28]. None of the SRs/MAs provided an exclusion of the reasons for each study, which may affect the reliability of the results. Providing a list of exclusion research can strongly demonstrate the rigor of the literature screening process. In addition, the lack of publication bias assessment may reduce the accuracy of the final results, which is also related to the insufficient number of RCTs included in the SRs/MAs. ROBIS is used to assess the risk of bias in the included SRs/MAs. Among them, the lack of sensitivity analysis and insufficient evaluation of publication bias are the main factors leading to a high risk of bias, which may affect the reliability of the final results. Similar to the results of the AMSTAR-2 and ROBIS assessments, the PRISMA assessment results indicate a lack of registration of programs and a lack of publication bias risk assessment. All of the above reporting deficiencies may affect the clarity and transparency of the SR/MA implementation method.

The quality of the evidence is assessed by the GRADE system. Among the 19 outcome indicators, the quality of evidence was 10 moderate, 6 low, and 3 very low. Imprecision is the most common degrading factor, followed by publication bias and inconsistency. Through further analysis, the total research population sample size of the RCTs included in the meta-analysis of the related outcome indicators is insufficient. This is an important reason for the insufficient quality of evidence. In addition, the publication bias analysis of the outcome indicators included in the SRs/MAs is not comprehensive, or there is a risk of publication bias, which can also lead to the unreliability of results.

Descriptive analysis shows that curcumin is an effective treatment for UC and may be safer than CT. However, there is a SR/MA that indicates that curcumin may be ineffective for UC patients. The reason may be that the SR/MA was published relatively early, and the inclusion of RCTs was relatively limited. Another possible explanation may stem from the highly heterogeneous oral curcumin doses observed in the retrieved RCTs. In addition, the administration of curcumin in SRs/Mas published later is not limited to oral administration. Due to the low methodological quality of the included studies, the conclusions of the SRs/MAs may be different from the real results, and caution should be taken when recommending curcumin as a supplementary intervention for UC.

### 4.2. Implications for Future Research

The AMSTAR-2, PRISMA, and ROBIS assessments can be used to evaluate all aspects of the included SRs/Mas, determine the outlook for the future SR/MA, and make it more standardized. Researchers should register or publish the research plan in advance when conducting SRs/Mas to avoid risk of bias and ensure the recognition of the SR/MA results. A list of excluded literature should be provided with explanations to ensure transparency and avoid publication bias. For literature with a high risk of bias, researchers should conduct a separate analysis and give a reasonable explanation to ensure the quality of the evidence. In addition, a complete evaluation of publication bias will also increase the accuracy of the meta-analysis results. The bioavailability of curcumin is low, resulting in lower plasma and tissue levels of curcumin. Therefore, in the next RCTs, researchers will explore better curcumin bioavailability by changing dosage forms, such as enema administration [29] and a self-microemulsifying drug delivery system [30]. With the development of evidence-based medicine, it is hoped that in the future, researchers will continue to promote the standardization of related single RCTs. Well-designed and strictly implemented RCTs can minimize or avoid bias. This is the gold standard for evaluating interventions [31].

### 4.3. Strengths and Limitations

As far as we know, our study is the first overview of SRs/Mas on the use of curcumin in the treatment of UC, which can provide a comprehensive evidence reference for clinical practice. Second, the evaluation process of AMSTAR-2, PRISMA, ROBIS, and GRADE revealed the obvious limitations of SRs/MAs and RCT, which may help guide high-quality research in the future. However, we must also acknowledge the limitations of this overview due to the generally low quality of SRs/MAs and outcome measures to draw firm conclusions. Caution is required in recommending curcumin as a complementary treatment for UC, and we only included SRs/MAs published in English and Chinese, so a small subset of studies in other languages may be missed.

## 5. Conclusion

According to the currently published evidence, curcumin may be effective and safe for the treatment of UC, especially in increasing clinical remission and improvement rate. However, due to the generally low quality of methodologies, reports, and evidence for the inclusion of SRs/MAs, caution should be exercised when recommending curcumin as a complementary treatment for UC. More standardized and comprehensive RCTs and SRs/MAs are needed to provide stronger evidence. In addition, it is also necessary to explore the multidosage form administration of curcumin in the application of UC.

## Figures and Tables

**Table 1:** Search strategy for the PubMed database.

#1	Curcumin OR “Turmeric Yellow” OR “Yellow, Turmeric” OR Diferuloylmethane
#2	“Colitis, Ulcerative”[MeSH]
#3	“chronic ulcerative colitis” OR “colitis ulcerativa” OR “colitis ulcerosa” OR “colitis ulcerosa chronica” OR “colitis, mucosal” OR “colitis, ulcerative” OR “colitis, ulcerous” OR “colon, chronic ulceration” OR “histiocytic ulcerative colitis” OR “mucosal colitis” OR “ulcerative colorectitis” OR “ulcerative procto colitis” OR “ulcerative proctocolitis” OR “ulcerous colitis” OR “ulcerative colitis”
#4	#2 AND #3
#5	Meta-Analysis as Topic [MeSH]
#6	“Systematic review” OR “meta-analysis” OR “meta analysis” OR “meta-analyses” OR “Review, systematic”
#7	#5 AND #6
#8	#1 AND #4 AND #7

**Table 2:** Characteristics of the included SRs/MAs.

Author, year (country)	Trials (subjects)	Intervention group	Control group	Mode of administration	Quality assessment	Main results
Saurabh Chandan, 2020 (USA) [20]	7 (380)	Curcumin+mesalamine	Placebo+mesalamine	Oral administration, enema administration	Jadad	According to our research, compared with placebo, the clinical remission rate of mesalazine and curcumin combined treatment is about 3 times higher, and the side effects are minimal. This response is statistically significant, although there is heterogeneity, it may be due to the severity score index, the dose of curcumin, and the route of administration used.
Armin Ebrahimzadeh, 2021 (Iran) [21]	2 (104)	Curcumin+CT	Placebo+CT	Oral administration	Cochrane	Curcumin supplementation is associated with a significant decrease in CRP and ESR levels in patients with ulcerative colitis.
Ricardo de Alvares Goulart, 2020 (Brazil) [22]	4 (238)	Curcumin+CT	Placebo+CT	Oral administration	GRADE	Based on our results, we can say that the use of curcumin has a beneficial effect on the clinical remission of UC patients. However, when treating UC, we need to consider curcumin carefully, because more robust and well-designed studies are needed.
Umair Iqbal, 2018 (USA) [23]	3 (142)	Curcumin+mesalamine	Placebo+mesalamine	Oral administration, enema administration	Jadad	This study shows that when curcumin is used in combination with mesalazine to achieve remission in UC patients, the clinical remission rate is higher. Because of its cost-effectiveness and safer side effects, curcumin can reduce the medical burden and morbidity associated with this recurrent and recurrent disease..
Maria G. Grammatikopoulou, 2018 (Greece) [24]	3 (194)	Curcumin+mesalamine	Placebo+mesalamine	Oral administration	Cochrane	Based on the currently available evidence, oral curcumin does not appear to be superior to placebo in alleviating the condition of UC patients.
Ting Zheng, 2020 (China) [25]	6 (349)	Curcumin+CT	Placebo+CT	Oral administration, enema administration	Cochrane	In short, curcumin is an effective and safe drug that can be used in the treatment of UC together with standard treatments.
Liwei Zhu, 2019 (China) [26]	5 (261)	Curcumin+CT	Placebo+CT	Oral administration, enema administration	Cochrane	Our systematic review and meta-analysis showed that curcumin combined with mesalazine as an adjuvant drug can significantly increase the clinical remission rate, DAI improvement rate, and mucosal healing rate of mesalamine.

**Table 3:** Result of the AMSTAR-2 assessments.

Author, year (country)	Q1	*Q2*	Q3	*Q4*	Q5	Q6	*Q7*	Q8	*Q9*	Q10	*Q11*	Q12	*Q13*	Q14	*Q15*	Q16	Quality
Saurabh Chandan, 2020 (USA) [20]	Y	PY	Y	Y	Y	Y	N	Y	Y	Y	Y	Y	Y	Y	N	Y	VL
Armin Ebrahimzadeh, 2021 (Iran) [21]	Y	PY	Y	Y	Y	Y	N	Y	Y	Y	Y	Y	Y	Y	N	Y	VL
Ricardo de Alvares Goulart, 2020 (Brazil) [22]	Y	PY	Y	PY	Y	Y	N	Y	Y	Y	Y	Y	Y	N	N	Y	VL
Umair Iqbal, 2018 (USA) [23]	Y	PY	Y	Y	Y	Y	N	Y	Y	Y	Y	Y	Y	Y	Y	Y	VL
Maria G. Grammatikopoulou, 2018 (Greece) [24]	Y	Y	Y	Y	Y	Y	N	Y	Y	Y	Y	Y	Y	Y	N	Y	VL
Ting Zheng, 2020 (China) [25]	Y	PY	Y	PY	Y	Y	N	Y	Y	Y	Y	Y	Y	Y	Y	Y	VL
Liwei Zhu, 2019 (China) [26]	Y	PY	Y	PY	Y	Y	N	Y	Y	Y	Y	Y	Y	Y	N	Y	VL

Note: Y, Yes; PY, partial Yes; N, No; VL, Very low; L, L. Note: Critical areas are marked in italic.

**Table 4:** Results of the ROBIS assessments.

Author, year (country)	Phase 1	Phase 2				Phase 3
	Assessing relevance	Domain 1: study eligibility criteria	Domain 2: identification and selection of studies	Domain 3: collection and study appraisal	Domain 4: synthesis and findings	Risk of bias in the review
Saurabh Chandan, 2020 (USA) [20]	√	√	√	√	×	√
Armin Ebrahimzadeh, 2021 (Iran) [21]	√	√	√	√	×	√
Ricardo de Alvares Goulart, 2020 (Brazil) [22]	√	√	×	×	×	√
Umair Iqbal, 2018 (USA) [23]	√	√	√	√	×	√
Maria G. Grammatikopoulou, 2018 (Greece) [24]	√	√	√	×	×	√
Ting Zheng, 2020 (China) [25]	√	√	×	×	×	√
Liwei Zhu, 2019 (China) [26]	√	√	×	×	×	√

Note: √, low risk; ×, high risk.

**Table 5:** Results of the PRISMA checklist.

Section/topic	Items	Saurabh Chandan, 2020 (USA) [20]	Armin Ebrahimzadeh, 2021 (Iran) [21]	Ricardo de Alvares Goulart, 2020 (Brazil) [22]	Umair Iqbal, 2018 (USA) [23]	Maria G. Grammatikopoulou, 2018 (Greece) [24]	Ting Zheng, 2020 (China) [25]	Liwei Zhu, 2019 (China) [26]	Number of yes (%)
Title	Q1.Title	Y	Y	Y	Y	Y	Y	Y	100%
Abstract	Q2. Structured summary	Y	Y	Y	Y	Y	Y	Y	100%
Introduction	Q3. Rationale	Y	Y	Y	Y	Y	Y	Y	100%
	Q4. Objectives	Y	Y	Y	Y	Y	Y	Y	100%
Methods	Q5. Protocol and registration	N	N	N	N	Y	N	N	14.30%
	Q6. Eligibility criteria	Y	Y	Y	Y	Y	Y	Y	100%
	Q7. Information sources	Y	Y	Y	Y	Y	Y	Y	100%
	Q8. Search	N	Y	Y	N	Y	Y	N	57.10%
	Q9. Study selection	Y	Y	Y	Y	Y	Y	Y	100%
	Q10. Data collection process	Y	Y	Y	Y	Y	Y	Y	100%
	Q11. Data items	Y	Y	Y	Y	Y	Y	Y	100%
	Q12. Risk of bias in individual studies	Y	Y	Y	Y	Y	Y	Y	100%
	Q13. Summary measures	Y	Y	Y	Y	Y	Y	Y	100%
	Q14. Synthesis of results	Y	Y	Y	Y	Y	Y	Y	100%
	Q15. Risk of bias across studies	N	N	N	Y	N	Y	N	28.60%
	Q16. Additional analyses	Y	Y	N	N	N	N	N	28.60%
Results	Q17. Study selection	Y	Y	Y	Y	Y	Y	Y	100%
	Q18. Study characteristics	Y	Y	Y	Y	Y	Y	Y	100%
	Q19. Risk of bias within studies	Y	Y	Y	Y	Y	Y	Y	100%
	Q20. Results of individual studies	Y	Y	Y	Y	Y	Y	Y	100%
	Q21. Synthesis of results	Y	Y	Y	Y	Y	Y	Y	100%
	Q22. Risk of bias across studies	N	N	N	Y	N	Y	N	28.60%
	Q23. Additional analysis	Y	Y	Y	Y	Y	Y	Y	100%
Discussion	Q24. Summary of evidence	Y	Y	Y	Y	Y	Y	Y	100%
	Q25. Limitations	Y	Y	N	Y	Y	Y	N	71.40%
	Q26. Conclusions	Y	Y	Y	Y	Y	Y	Y	100%
Funding	Q27. Funding	Y	N	Y	Y	N	Y	N	57.10%

Note: Y, yes; N, no.

**Table 6:** Results of evidence quality.

Author, year (country)	Outcomes	Studies (participants)	Limitations	Inconsistency	Indirectness	Imprecision	Publication bias	Relative effect (95% CI)	Heterogeneity	Quality
Saurabh Chandan, 2020(USA) [20]	Clinical remission rate	5 (282)	0	0	0	0	-1④	OR: 2.9 (95% CI: 1.5, 5.5)∗	I^2^ = 45%	Moderate
	Clinical improvement rate	5 (255)	0	0	0	0	-1④	OR: 2.6 (95% CI: 1.5, 4.5)∗	I^2^ = 74%	Moderate
	Summary rate of endoscopic improvement and remission	5 (235)	0	0	0	0	-1④	OR: 2.3 (95% CI: 1.2, 4.6)∗	I^2^ = 35.5%	Moderate
Armin Ebrahimzadeh, 2021(Iran) [21]	CRP	1 (63)	0	-1②	0	-1③	-1④	WMD: -0.15 (95% CI: -0.28, -0.02)∗	NA	Very low
	ESR	2 (104)	0	0	0	-1③	-1④	WMD: -6.92 (95% CI: -11.83, -2)∗	I^2^ = 59.3%	Low
Ricardo de Alvares Goulart, 2020(Brazil) [22]	Clinical remission rate	3 (182)	0	-1②	0	-1③	-1④	RD: 0.31 (95% CI: 0.02, 0.60)∗	I^2^ = 82%	Very low
	Clinical improvement rate	3 (182)	0	-1②	0	-1③	-1④	RD: 0.24 (95% CI: -0.15, 0.63)	I^2^ = 90%	Very low
Umair Iqbal, 2018(USA) [23]	Clinical remission rate	2 (95)	0	-1②	0	-1③	0	OR: 6.78 (95% CI: 2.39, 19.23)∗	I^2^ = 75.9%	Low
	Clinical improvement rate	3 (142)	0	0	0	-1③	0	OR: 4.65 (95% CI: 2.18, 9.92)∗	I^2^ = 40.7%	Moderate
	Endoscopic improvement rate	2 (95)	0	0	0	-1③	0	OR: 3.82 (95% CI: 1.40, 10.40)∗	I^2^ = 63.7%	Moderate
	Endoscopic remission rate	2 (102)	0	0	0	-1③	0	OR: 12.74 (95% CI: 1.56, 104.07)∗	I^2^ = 0%	Moderate
Maria G. Grammatikopoulou, 2018(Greece) [24]	Clinical remission rate	3 (201)	0	0	0	0	-1⑤	OR: 3.80 (95% CI: 0.55,26.28)	I^2^ = 63.7%	Moderate
Ting Zheng, 2020(China) [25]	Clinical remission rate	4 (198)	0	0	0	-1③	0	OR:5.18 (95% CI: 1.84, 14.56)∗	I^2^ = 33%	Moderate
	Endoscopic remission rate	3 (121)	0	0	0	-1③	-1⑤	OR: 5.69 (95% CI:1.28, 25.27)∗	I^2^ = 28%	Low
	Clinical improvement rate	4 (158)	0	0	0	-1③	0	OR:4.79 (95% CI: 1.02, 22.43)∗	I^2^ = 75%	Moderate
	Endoscopic improvement rate	2 (71)	0	0	0	-1③	0	OR:17.05 (95% CI:: 1.30, 233.00)∗	I^2^ = 57%	Moderate
Liwei Zhu, 2019(China) [26]	Clinical remission rate	3 (181)	0	0	0	-1③	-1④	OR: 4.78 (95% CI:1.24, 18.47)∗	I^2^ = 52%	Low
	Clinical improvement rate	3 (142)	0	0	0	-1③	-1④	OR: 4.61 (95% CI:2.22, 9.57)∗	I^2^ = 28%	Low
	Endoscopic remission rate	3 (142)	0	0	0	-1③	-1④	OR: 4.58 (95% CI:1.79, 11.73)∗	I^2^ = 49%	Low

Note: ①The included studies have a large bias in methodology such as randomization, allocation concealment, and blinding. ②The confidence interval overlaps less or the I2 value of the combined results was larger. ③The sample size from the included studies does not meet the optimal sample size or the 95% confidence interval crosses the invalid line. ④The funnel chart is asymmetry. ⑤Few studies were included, and their results were all positive, which may result in a large publication bias; ∗The 95% confidence interval does not cross the invalid line.

## Data Availability

The datasets analysed during the current study are available from the corresponding author on reasonable request.

## Abbreviations

UC: Ulcerative colitis
SR: Systematic review
MA: Meta-analysis
RCT: Randomized controlled trial
CT: Conventional treatment
AMSTAR-2: Assessment System for Evaluating Methodological Quality 2
ROBIS: Risk of Bias in Systematic reviews
PRISMA: Preferred Reporting Items for Systematic Reviews and Meta-Analyses
GRADE: Grading of Recommendations Assessment, Development, and Evaluation
CAI: Clinical Activity Index
UCDAI: Ulcerative Colitis Disease Activity Index
SCCAI: Simple Clinical Colitis Activity Index
CRP: C-reactive protein
ESR: Erythrocyte sedimentation rate.
